# The validation of the Dutch SF-Qualiveen, a questionnaire on urinary-specific quality of life, in spinal cord injury patients

**DOI:** 10.1186/s12894-017-0280-9

**Published:** 2017-09-19

**Authors:** Sarah H. M. Reuvers, Ida J. Korfage, Jeroen R. Scheepe, Lisette A. ‘t Hoen, Tebbe A. R. Sluis, Bertil F. M. Blok

**Affiliations:** 1 Department of Urology, Erasmus MC, Wijtemaweg 80, Room Na 1724, 3015 CN Rotterdam, The Netherlands; 2 Department of Public Health, Erasmus MC, Rotterdam, The Netherlands; 3 Department of Rehabilitation, Rijndam Rehabilitation, Rotterdam, The Netherlands

**Keywords:** Patient reported outcome measure, Urinary bladder, neurogenic, Validation studies, Quality of life, Surveys and questionnaires

## Abstract

**Background:**

Optimizing the patients’ quality of life is one of the main goals in the urological management of spinal cord injury (SCI) patients. In this study we validated the Dutch SF-Qualiveen, a short questionnaire that measures the urinary-specific quality of life, in SCI patients. No such measure is yet available for this patient group.

**Methods:**

In 2015–2016 SCI patients with urinary symptomatology who visited the outpatient clinics of Urology at the Erasmus Medical Centre and Rehabilitation at Rijndam Revalidation completed the SF-Qualiveen and UDI-6 during the visit and 1–2 weeks later. The UDI-6, a urinary tract symptom inventory, served as gold standard. Controls, recruited from the Otolaryngology outpatient clinic, completed the questionnaires once. Content-, construct-, and criterion validity and reliability (internal consistency and reproducibility) of the SF-Qualiveen were determined.

**Results:**

Fifty seven SCI patients and 50 controls were included. 12 SCI patients asserted that the SF-Qualiveen covered their bladder problems (good content validity). Patients’ SF-Qualiveen scores being positively associated with severity of urinary symptoms and patients’ scores being higher than those of controls indicated good construct validity. The positive association that was found between SF-Qualiveen and UDI-6 in patients (*r* = 0.66–0.67, *P* < 0.001) and controls (*r* = 0.63, P < 0.001) confirmed good criterion validity. Internal consistency (Cronbach’s alpha 0.89–0.92) and reproducibility (intraclass correlation coefficient 0.94) of the SF-Qualiveen were good.

**Conclusions:**

The Dutch SF-Qualiveen is a valid and reliable tool to measure the urinary-specific quality of life in SCI patients.

## Background

Spinal cord injury (SCI) causes urological dysfunction in 70–84% of patients [1]. The type of detrusor and/or sphincter dysfunction depends on the localization of the SCI and the damage to the spinal cord. Clinical presentation can vary from urinary incontinence to inability to empty the bladder [2].

More than two thirds of SCI patients in the Netherlands reported bladder regulation problems as one of their most frequent health problems [3]. Bladder problems were perceived as a major secondary impairment and as having the greatest impact on social life [3]. Bladder problems in patients with SCI were found to be associated with a lower quality of life [4]. Optimizing the quality of life is considered one of the most important aspects in the urological management of patients with neuro-urological dysfunction due to SCI [5].

Currently, there is no validated measure available in the Netherlands to evaluate the urinary-specific quality of life in SCI patients. The Qualiveen-30 [6] and its short version, the SF-Qualiveen [7], are measures that evaluate urinary-specific quality of life in patients with neurological disorders. The Qualiveen-30 has been validated in both multiple sclerosis (MS) and SCI patients [6, 8], but is not available in Dutch. Based on data of MS patients only, the eight most responsive items of the Qualiveen-30 were used to create the SF-Qualiveen [7]. The SF-Qualiveen has been validated in English [7], French [7] and Dutch [9] for MS patients, but not yet for SCI patients. Although the neuro-urological dysfunction in MS and SCI patients is similar in some aspects, its clinical presentation and the influence on the quality of life might differ due to dissimilarities between the two diseases (e.g. the onset of disease is acute in SCI vs. progressive in MS; SCI often entails a total loss of sensation of the lower body, while MS entails an altered sensibility, but often no total loss of sensibility). For this reason, it is essential to evaluate the validity and reliability of the SF-Qualiveen in SCI patients before its use can be recommended as a measurement tool in the management of Dutch SCI patients to optimize their quality of life.

## Methods

### Design and subjects

The research protocol (MEC-2014-534) was reviewed by the local medical research ethics committee, which concluded that the rules as stated in the Dutch Medical Research Involving Human Subjects Act did not apply to this study. The study was conducted at the Urology outpatient clinic of the Erasmus University Medical Center (Erasmus MC), Rotterdam, the Netherlands and at the Rehabilitation outpatient clinic at Rijndam Rehabilitation, Rotterdam, the Netherlands. In August and September 2015 face-to-face interviews were conducted with SCI patients with urinary symptomatology to assess content-validity of the Dutch translated version of the SF-Qualiveen. Between late September 2015 and May 2016 adult patients with SCI and urinary symptomatology were included. We intended to invite all eligible consecutive patients who visited the outpatients clinics to participate. Exclusion criteria were cognitive impairment, Dutch language difficulties, recent malignant tumors, symptomatic urinary tract infections, and (foreseen) change of (bladder-specific) treatment within the test-retest period. After having provided written informed consent, participants completed the SF-Qualiveen and the Urinary Distress Inventory-6 (UDI-6) at the outpatient clinic (test) and 1 to 2 weeks later at home (re-test). Clinical characteristics of included patients were retrieved from their medical charts.

We used earlier collected data of a control group, that was recruited at the Otolaryngology outpatient clinic in 2016 [9]. Exclusion criteria for this group were cognitive impairment, Dutch language difficulties and neuro-urological dysfunction. The control patients had provided written informed consent and completed the measures once.

### Measures

The SF-Qualiveen is a measure that evaluates the urinary-specific quality of life in neuro-urological patients. Table 1 shows the eight questions of the questionnaire. Each item is scored on an ordinal Likert scale ranging from 0 (no impact) to 4 (high impact). The total score is the mean of the eight separate scores [7]. The SF-Qualiveen consists of four domains, each containing two questions: bother with limitations (question 1 and 2), fears (question 3 and 4), feelings (question 5 and 6) and frequency of limitations (question 7 and 8).

**Table 1:** Questions of the SF-Qualiveen

1. In general, do your bladder problems complicate your life?	
2. Are you bothered by the time spent passing urine or realizing catheterization?	
3. Do you worry about your bladder problems worsening?	
4. Do you worry about smelling of urine?	
5. Do you feel worried because of your bladder problems?	
6. Do you feel embarrassed because of your bladder problems?	
7. Is your life regulated by your bladder problems?	
8. Can you go out without planning anything in advance?	

The Dutch UDI-6 is a validated Dutch measure [10], but has not been specifically validated in a neuro-urological patient group. The questionnaire (six questions) assesses the severity of urinary tract symptoms. It consists of three domains: irritative, stress and obstructive/discomfort urinary symptoms [11]. We chose this measure as a gold standard in the absence of a perfect gold standard for this patient group.

### Validation process

The cross-cultural adaptation of the SF-Qualiveen into Dutch by our group was previously described [9]. In short; two forward-translations of the SF-Qualiveen from English to Dutch, and one backward translation were followed by consensus meetings between translators and clinicians. Standardized guidelines for linguistic validation were followed [12]. *Content validity* was assessed by face-to-face interviews with SCI and MS patients [13]. The goal of these interviews was to confirm that the translated version of the SF-Qualiveen used clear wording and that it was a complete measure.

In the current study, predefined hypotheses on *construct validity* were assessed:

1. We hypothesized that SF-Qualiveen scores of patients would be positively associated with the severity of urinary symptoms (UDI-6 domains irritative, stress and obstructive/discomfort urinary symptoms and total score).
2. We hypothesized that scores of the SF-Qualiveen in the patient group would be higher than scores in the control group.

*Criterion validity* was determined by assessing the relationship between the SF-Qualiveen and the UDI-6 as a gold standard. *Floor and ceiling effects* were presumed to be present if more than 15% of respondents achieved the highest or lowest possible score. Therefore, percentages of respondents with the highest and lowest possible score were calculated. A floor effect was to be expected in the control group.

The *internal consistency* of the SF-Qualiveen questions*,* i.e. whether the questions measure the same underlying construct, was determined by calculating Cronbach’s alpha. The *reproducibility* of the SF-Qualiveen was determined by calculating the intraclass correlation coefficient (ICC) for agreement of the repeated measurements. The *limits of agreement* (LOA) were determined. In general, differences in scores within the LOA can be interpreted as measurement error [14].

A post hoc subgroup analysis was performed to investigate construct- and criterion validity, internal consistency and reproducibility of the Dutch SF-Qualiveen in different subgroups based on level of SCI, ASIA (American Spinal Injury Association) Impairment Scale and manner of bladder emptying.

### Statistical analyses

We aimed to include at least 50 patients and 50 controls to comply with the guidelines for validation of questionnaires [13]. For the face-to-face interviews we aimed to include at least 10 SCI patients.

For the statistical analyses we used SPSS version 21. Descriptive results are presented as mean ± standard deviations for continuous data and counts and percentages for discrete data. Student’s T-tests were used to assess differences between groups for continuous variables and Chi-Square tests for categorical variables. Associations between variables were assessed using the Pearson’s correlation coefficient in case of a linear association. Cronbach’s alpha’s were calculated to determine the internal consistency. Cronbach’s alpha’s between 0.7 and 0.95 were considered good [13]. The LOA were calculated as the mean change in scores of repeated measurements ±1.96 x standard deviation (SD) of the changes [14]. ICCs of 0.7 or higher were considered to represent good reproducibility [13]. Statistical significance was assumed at a *p*-value of less than 0.05.

## Results

66 SCI patients completed the questionnaires at baseline (‘test’). Seven patients did not return the second questionnaires while one declined further participation. The mean SF-Qualiveen score (test) of these patients was 1.81 ± 0.65. One patient was diagnosed with a malignant tumor and excluded. In total, 57 SCI patients completed the second questionnaires (retest) on average 12.7 (±9.0) days after the first questionnaires and were included in the analyses. Characteristics of the study groups are displayed in Table 2. Most patients had a thoracic SCI, required a wheelchair for mobility and were dependent upon catheterization (intermittent or indwelling) to empty their bladder. The 50 controls were significantly younger than the SCI patients. The proportion of males and females was similar in both groups.

### Validation process

Following the translation of the SF-Qualiveen into Dutch, 12 SCI patients and 11 MS patients were interviewed to assess c*ontent validity*. The translated SF-Qualiveen was distributed to the patients. Thereafter, patients were asked whether the questions covered all the bladder problems that affected their quality of life. Both patient groups agreed on the importance of the questions and found it a complete measure that covered the broad range of bladder problems that they experienced. Furthermore, patients found the Dutch version clear and easy to complete.

**Table 2:** Clinical characteristics

		Patients	Controls	*P*-value
N		57	50	
Age at examination		53.2 ± 14.6	42.3 ± 14.2	<0.001
Sex	Male	37 (64.9%)	26 (52.0%)	0.176
	Female	20 (35.1%)	24 (48.0%)	
Years after SCI		13.1 ± 12.8		
Level of SCI	Cervical	15 (26.3%)		
	Thoracic	31 (54.4%)		
	Lumbar	11 (19.3%)		
ASIA Impairment Scale	A	23 (40.3%)		
	B	5 (8.8%)		
	C	7 (12.3%)		
	D	20 (35.1%)		
		Missing: 2 (3.5%)		
Mobility	Fully ambulatory	4 (7.0%)		
	Limited walking	16 (28.1%)		
	Wheelchair only	35 (61.4%)		
		Missing: 2 (3.5%)		
Manner of bladder emptying	(normal) voiding	5 (8.8%)		
	Abdominal pressure	1 (1.8%)		
	Total incontinence	1 (1.8%)		
	Intermittent catheterization	27 (47.4%)		
	Indwelling catheter	22 (38.6%)		
		Missing: 1 (1.8%)		

Results are presented as mean ± standard deviations for continuous data and counts and percentages for discrete data. ASIA Impairment Scale, American Spinal Injury Association Impairment Scale (A: Complete, B: Sensory incomplete, C: Motor incomplete - half of key muscle functions below the neurological level of injury have a muscle grade less than 3, D: Motor incomplete - at least half of key muscle functions below the neurological level of injury have a muscle grade > 3) [16]; SCI, Spinal Cord Injury

The predefined hypotheses on *construct validity* were confirmed:

1. Positive significant associations were found between both the total UDI-6 and the different domains of the UDI-6 which measure the severity of irritative, stress and obstructive/discomfort urinary symptoms and the total SF-Qualiveen scores in the patient group. (Table 3) The hypothesis that SF-Qualiveen scores of patients would be positively associated with the severity of urinary symptoms was confirmed.
2. The mean of the total scores of the SF-Qualiveen for the patient group was 1.81 ± 0.99 for the test and 1.80 ± 1.08 for the re-test while the control group reported a mean score of 0.34 ± 0.59 (*P* < 0.001). In an older subgroup of controls >40 years (*n* = 27, mean age 53.9 years) the mean total SF-Qualiveen score was 0.51. A significant difference in mean SF-Qualiveen scores between the patient group and the control group >40 years was found (P < 0.001).

**Table 3:** Correlations between severity of urinary symptoms (UDI-6 domain scores) – and SF-Qualiveen total scores in patient group

	Test	Re-test
UDI-6 – total score	*r* = 0.663 and P < 0.001	*r* = 0.673 and *P* < 0.001
Severity of irritative urinary symptoms	*r* = 0.596 and *P* < 0.001	*r* = 0.543 and P < 0.001
Severity of stress urinary symptoms	*r* = 0.451 and P < 0.001	*r* = 0.424 and *P* = 0.001
Severity of obstructive/discomfort urinary symptoms	*r* = 0.521 and P < 0.001	*r* = 0.630 and P < 0.001

Pearson’s correlation coefficients were determined to assess the relationship between variables. UDI-6, Urinary Distress Inventory-6

A significant positive association between the SF-Qualiveen and the UDI-6 was found in both the patient (Table 3) and control group (*r* = 0.632 and P < 0.001). *Criterion validity* was hereby found to be good. *Floor and ceiling effects* were not found in the patient group for the total SF-Qualiveen score (Test: no patients had the lowest or highest possible score. Re-test: 2% of the patients had the lowest and 2% had the highest possible score). As expected, a floor effect was found in the control group for the total SF-Qualiveen score: 50% of the controls had the lowest possible score. No ceiling effect was found in the control group (none had the highest possible score).

Cronbach’s alpha’s of 0.89 (test) and 0.92 (re-test) indicated good *internal consistency* for the total SF-Qualiveen. (Table 4) The domains ‘bother with limitations’ and ‘feeling’ showed good internal consistency as well. Internal consistency of the domains ‘fears’ and ‘frequency of limitations’ was moderate. The ICCs for the repeated measurements of the test and re-test for the SF-Qualiveen total score and domain scores showed good *reproducibility* (Table 5). Table 5 shows the *limits of agreement* (LOA) as well. Differences between −0.72 and 0.70 can be interpreted as not clinically important.

**Table 4:** Internal consistency – Cronbach’s alpha (*n* = 57 SCI patients)

	Test	Re-test
SF-Qualiveen total score	0.89	0.92
SF-Qualiveen domains:		
Bother with limitations	0.87	0.90
Fears	0.53	0.73
Feeling	0.80	0.84
Frequency of limitations	0.55	0.75

*SCI* Spinal Cord Injury

**Table 5:** Reproducibility of SF-Qualiveen

	ICC	LOA
SF-Qualiveen total score	0.94	−0.72 to 0.70
Bother with limitations	0.90	−1.12 to 1.00
Fears	0.92	−0.97 to 0.99
Feeling	0.87	−1.27 to 1.23
Frequency of limitations	0.79	−0.72 to 0.70

*ICC* Intraclass Correlation Coefficient, *LOA* Limits of Agreement

In Table 6 the results of the post hoc subgroup analyses based on level of SCI, ASIA Impairment Scale and manner of bladder emptying are shown. Most subgroups showed a positive significant association between the SF-Qualiveen total scores and the UDI-6 score and a significant difference in mean SF-Qualiveen scores compared to the control group, indicating good criterion and construct validity. Cronbach’s alpha’s of >0.79 and ICCs >0.86 confirmed good internal consistency and reproducibility for the different subgroups.

**Table 6:** Subgroup analyses

		Patient numbers	Mean total SF-Qualiveen scores	Cronbach’s alpha	ICC	Correlation between SF-Qualiveen scores and UDI-6 scores	Patients’ SF-Qualiveen scores compared to controls
Level of SCI	Cervical	15 (26%)	1.68–1.68	0.93–0.96	0.95	*r* = 0.853, *p* < 0.001 *r* = 0.788, *p* < 0.001	p < 0.001
	Thoracic	31 (55%)	1.77–1.75	0.88–0.91	0.95	*r* = 0.552, p = 0.001 *r* = 0.547, *p* = 0.001	p < 0.001
	Lumbar	11 (19%)	2.11–2.15	0.79–0.80	0.89	*r* = 0.686, p = 0.02 *r* = 0.769, *p* = 0.006	p < 0.001
ASIA Impairment Scale	A	23 (40.3%)	1.58–1.50	0.88–0.92	0.94	*r* = 0.585, *p* = 0.003 *r* = 0.650, *p* = 0.001	p < 0.001
	B	5 (8.8%)	2.18–2.48	0.80–0.88	0.92	*r* = 0.895, *p* = 0.040 *r* = 0.597, *p* = 0.287	p < 0.001
	C	7 (12.3%)	1.70–1.57	0.83–0.82	0.86	*r* = 0.715, *p* = 0.071 *r* = 0.677, *p* = 0.095	p < 0.001
	D	20 (35.1%)	2.04–2.11	0.90–0.92	0.96	*r* = 0.706, p < 0.001 *r* = 0.709, *p* < 0.001	p < 0.001
		Missing: 2					
Manner of bladder emptying	No catheter use	7 (12%)	1.36–1.21	0.87–0.91	0.94	*r* = 0.707, *p* = 0.076 *r* = 0.817, *p* = 0.025	p < 0.001
	Intermittent catheterization	27 (47%)	2.19–2.20	0.89–0.88	0.92	*r* = 0.571, *p* = 0.002 *r* = 0.518, *p* = 0.006	p < 0.001
	Indwelling catheter	22 (39%)	1.47–1.48	0.85–0.93	0.95	*r* = 0.743, p < 0.001 *r* = 0.768, p < 0.001	p < 0.001
		Missing: 1					

*ASIA Impairment Scale* American Spinal Injury Association Impairment Scale, *ICC* Intraclass Correlation Coefficient

## Discussion

In this study we introduced the SF-Qualiveen in a SCI patient group. We showed good content-, construct- and criterion validity, internal consistency and reproducibility of the SF-Qualiveen in this patient group. We conclude that the SF-Qualiveen can be used in the Netherlands to evaluate the urinary-specific quality of life in SCI patients.

The ICCs of the repeated measurements in this study (ranging from 0.79 to 0.94) showed good reproducibility for the total SF-Qualiveen and the separate domains, although they were somewhat lower than the ICCs found in the French and English SF-Qualiveen validation study in MS patients (0.88 to 0.94) [7]. The ICCs as found in the present study are comparable to the Dutch validation study of the SF-Qualiveen in MS patients (0.72 to 0.90) [9]. The Dutch SF-Qualiveen showed to be a reliable instrument for SCI patients.

Internal consistency for the total SF-Qualiveen was good. Cronbach’s alpha’s of 0.53 to 0.75 for the separate domains ‘fears’ and ‘frequency of limitations’ showed moderate internal consistency. This is consistent with results from the Dutch validation study of the SF-Qualiveen in MS patients [9]. Internal consistency was not described in the French and English validation study of the SF-Qualiveen. These study results indicate that the four domains of the Qualiveen-30 cannot be confirmed in the SF-Qualiveen, probably due to the small number of questions (two) in every domain. This strengthens the previous recommendation of Reuvers et al. [9] to not use the separate domains of the SF-Qualiveen, but only the total SF-Qualiveen.

The results of the subgroup analyses suggest that the Dutch SF-Qualiveen has equal measurement properties for SCI patients with different levels of SCI, ASIA Impairment statuses and manners of bladder emptying. Not finding a statistical significant correlation between the SF-Qualiveen scores and UDI-6 scores in the ASIA group B (*n* = 5) and C (*n* = 7) and the group without catheter usage (n = 7) could be explained by the lack of statistical power in the small patient groups due to the post hoc analysis.

Most SCI patients experience bladder problems as a consequence of damage to the spinal cord [1, 3]. These bladder problems have a negative effect on patients’ quality of life [4]. In the urological management of SCI patients optimization of the quality of life is an important aspect as mentioned in the EAU guidelines [5]. Therefore, it is essential for healthcare professionals to be informed about a patients’ present urinary-specific quality of life. The SF-Qualiveen is now available to objectively assess this topic in the Dutch SCI population. Only after being informed about present urinary-specific quality of life, an optimal treatment plan can be defined.

For the future we suggest that urology and rehabilitation departments in the Netherlands implement the Dutch-version SF-Qualiveen in the urological management of SCI patients. The Dutch SF-Qualiveen is now available as a measurement tool. Further research should be aimed at determining its responsiveness to treatment. Once this has been established as sufficient, the Dutch SF-Qualiveen may be used to evaluate the effect of treatments on the urinary-specific quality of life in clinical and research settings.

A question that arises is if we can recommend the use of the SF-Qualiveen in all neuro-urological patients. D’Ancona et al. [15] included, next to 33 SCI and eight MS patients, 10 patients with meningomyelocele (MMC) in the validation study of the Portuguese Qualiveen-30. Results of the different patient groups were not separately described. The authors state that MMC patients would have the same concerns regarding urinary-specific quality of life as SCI and MS patients. However, there might be a difference in the experience of patients with congenital neurological diseases such as MMC compared to patients with acquired diseases like SCI and MS. Therefore, it would be valuable to study the usefulness of the SF-Qualiveen in congenital neurological patients.

It is questionable if our Dutch version SF-Qualiveen validated in the Netherlands can be used in other Dutch speaking countries such as Belgium and South-Africa. Although the language is technically the same, wording and expressions can be different as well as cultural habits. Therefore, we recommend a new validation process before introducing the Dutch SF-Qualiveen in other Dutch language countries.

A strength of this study was the homogeneous patient group of SCI patients. Study results therefore provide a clear view of the validity and reliability of the SF-Qualiveen in this patient group. Furthermore, as this study was conducted at the outpatient clinics of urology of a general hospital and rehabilitation clinic, the SF-Qualiveen may be considered suitable for the use in both settings.

A limitation of the study was that eight of 66 patients (12.1%) were excluded because they did not complete the second questionnaire. This may have introduced a selection bias. However, the SF-Qualiveen scores (test) of these patients were similar to those of the included patients. Therefore, the selection bias may not be an important issue. Another limitation is that no other validated urinary-specific quality of life measure for neuro-urological patients is available to serve as a perfect gold standard to determine the criterion validity of the SF-Qualiveen. In the absence of a perfect gold standard, we chose the UDI-6, a urinary tract symptom inventory, which may have been suboptimal. In addition, criticism could be raised on the age difference between the patient and control group. To investigate one of the hypotheses on construct validity, we used data of a control group. We hypothesized that scores of the SF-Qualiveen in the patient group would be higher than scores in the control group. As a consequence of using earlier collected data, the age of the patient and control group were not matched and we found a statistical significant age difference between the groups. However, we did not expect this age difference to influence outcomes. We assumed that non-neuro-urological patients of the control group, regardless of their age, would have lower scores on a measure that evaluates the urinary-specific quality of life (developed for the use in neuro-urological patients) than the neuro-urological patient group. This expectation was strengthened by the fact that we also found a statistical significant difference in SF-Qualiveen scores between the patient group and the older control group (>40 years).

## Conclusions

From this study we can conclude that the Dutch SF-Qualiveen is valid and reliable to measure the urinary-specific quality of life in SCI patients. This short questionnaire, which is easy to complete, can be a valuable instrument. We suggest to use the total Dutch SF-Qualiveen for evaluation of the urinary-specific quality of life in SCI patients.

## Abbreviations

Erasmus MC: Erasmus University Medical Center
ICC: Intraclass correlation coefficient
LOA: Limits of agreement
MMC: Meningomyelocele
MS: Multiple sclerosis
SCI: Spinal cord injury
SD: Standard deviation
SUI: Stress urinary incontinence
UI: Urinary incontinence
